# Supplementary material for: Overcoming Anxiety Disorder by Probiotic Lactiplantibacillus plantarum LZU-J-TSL6 through Regulating Intestinal Homeostasis
Source: Foods. 2022 Nov 11;11(22):3596. doi: 10.3390/foods11223596 (PMC9689226; doi:10.3390/foods11223596)
Supplement: Supplementary file 1 [file foods-11-03596-s001.zip › foods-1963443-supplementary.pdf]

Supplementary Table S1. Reagent preparation list

The name of the reagent	usage amount
5×gDNA Eraser Buffer	2.0 μl
gDNA Eraser	1.0 μl
Total RNA	X μl
RNase-free H2O	7-X μl
Total	10 μl

Supplementary Table S2. List of reagents for the SYBR Green qPCR assay

The name of the reagent	usage amount
Step one Reaction solution	10 μl
PrimerScript Enzyme Mix 1	1.0 μl
RT Primer Mix	1.0 μl
5×PrimerScript Buffer	4.0 μl
RNase-free H2O	4.0 μl
Total	20 μl

Supplementary Table S3. primer sequence

Primer	Sequence	PCR products（bp）
Nrf2-F	TAGATGACCATGAGTCGCTTGC	153 bp
Nrf2-R	GCCAAACTTGCTCCATGTCC	
ZO1-F	AGAGATGAGCGGGCTACCTTACTG	132 bp
ZO1-R	GTCATGCGAGCGACCTGAATGG	

Supplementary Table S4. qPCR reaction system

Reagent	usage amount
cDNA template	1 μl
Primer（F+R）	1 μl
2×qPCR Mix	10 μl
Sterile water	8 μl

Supplementary Table S5. Sequencing sequence table of strain *LZU-J-TSL6*

**ATGGTGTGCTATGATGCAGTCGACGAACTCTGGTATTGATTGGTGCTTGCATCA**
**TGATTTACATTTGAGTGAGTGGCGAACTGGTGAGTAACACGTGGGAAACCTGC**
**CCAGAAGCGGGGGATAACACCTGGAAACAGATGCTAATACCGCATAACAACTT**
**GGACCGCATGGTCCGAGCTTGAAAGATGGCTTCGGCTATCACTTTTGGATGGTC**
**CCGCGGCGTATTAGCTAGATGGTGGGGTAACGGCTCACCATGGCAATGATACG**
**TAGCCGACCTGAGAGGGTAATCGGCCACATTGGGACTGAGACACGGCCCAAAC**
**TCCTACGGGAGGCAGCAGTAGGGAATCTTCCACAATGGACGAAAGTCTGATGG**
**AGCAACGCCGCGTGAGTGAAGAAGGGTTTCGGCTCGTAAAACTCTGTTGTTAA**
**AGAAGAACATATCTGAGAGTAACTGTTCAGGTATTGACGGTATTTAACCAGAA**
**AGCCACGGCTAACTACGTGCCAGCAGCCGCGGTAATACGTAGGTGGCAAGCGT**
**TGTCCGGATTTATTGGGCGTAAAGCGAGCGCAGGCGGTTTTTTAAGTCTGATGT**
**GAAAGCCTTCGGCTCAACCGAAGAAGTGCATCGGAAACTGGGAAACTTGAGTG**
**CAGAAGAGGACAGTGGAACTCCATGTGTAGCGGTGAAATGCGTAGATATATGG**
**AAGAACACCAGTGGCGAAGGCGGCTGTCTGGTCTGTAACTGACGCTGAGGCTC**
**GAAAGTATGGGTAGCAAACAGGATTAGATACCCTGGTAGTCCATACCGTAAAC**
**GATGAATGCTAAGTGTTGGAGGGTTTCCGCCCTTCAGTGCTGCAGCTAACGCAT**
**TAAGCATTCCGCCTGGGGAGTACGGCCGCAAGGCTGAAACTCAAAGGAATTGA**
**CGGGGGCCCGCACAAGCGGTGGAGCATGTGGTTTAATTCGAAGCTAC**
